# Evaluating Payments for Environmental Services: Methodological Challenges

**DOI:** 10.1371/journal.pone.0149374

**Published:** 2016-02-24

**Authors:** Gwenolé Le Velly, Céline Dutilly

**Affiliations:** 1 CERDI, Université d’Auvergne, Clermont-Ferrand, France; 2 SELMET, CIRAD, Montpellier, France; University of Vermont, UNITED STATES

## Abstract

Over the last fifteen years, Payments for Environmental Services (PES) schemes have become very popular environmental policy instruments, but the academic literature has begun to question their additionality. The literature attempts to estimate the causal effect of these programs by applying impact evaluation (IE) techniques. However, PES programs are complex instruments and IE methods cannot be directly applied without adjustments. Based on a systematic review of the literature, this article proposes a framework for the methodological process of designing an IE for PES schemes. It revises and discusses the methodological choices at each step of the process and proposes guidelines for practitioners.

## 1 Introduction

Many authors have recently called for more rigorous impact evaluation (IE) of conservation instruments [1–5]. As pointed out by Baylis et al. (2015), given the scarcity of financial resources, it is crucial to understand how and why programs succeed or fail to achieve their objectives. However, evaluating the impact of a Payments for Environmental Services (PES) scheme goes beyond monitoring indicators and involves estimating the additionality by isolating a causal effect of the scheme on the outcome of interest.

PES schemes have become very popular tools for the preservation and restoration of ecosystem services. Based on the assumption that environmental problems come from an under-provision of environmental services, PES schemes are defined by Wunder (2015) [6] as *≪ voluntary transactions between service users and service providers that are conditional on agreed rules of natural resource management for generating offsite services ≫*.

IE methods for PES schemes are inspired by former studies on conditional cash transfers in other fields of economics such as health or education economics [7]. These studies estimate the impact of a policy intervention by comparing a group of beneficiaries, the treatment group, and a group of non beneficiaries, the control group. The rationale for IE methodologies is to take into account the selection bias between the treatment and the control group through techniques such as matching or difference-in-difference in order to identify the causal effect of the treatment on an outcome.

**Fig 1:**
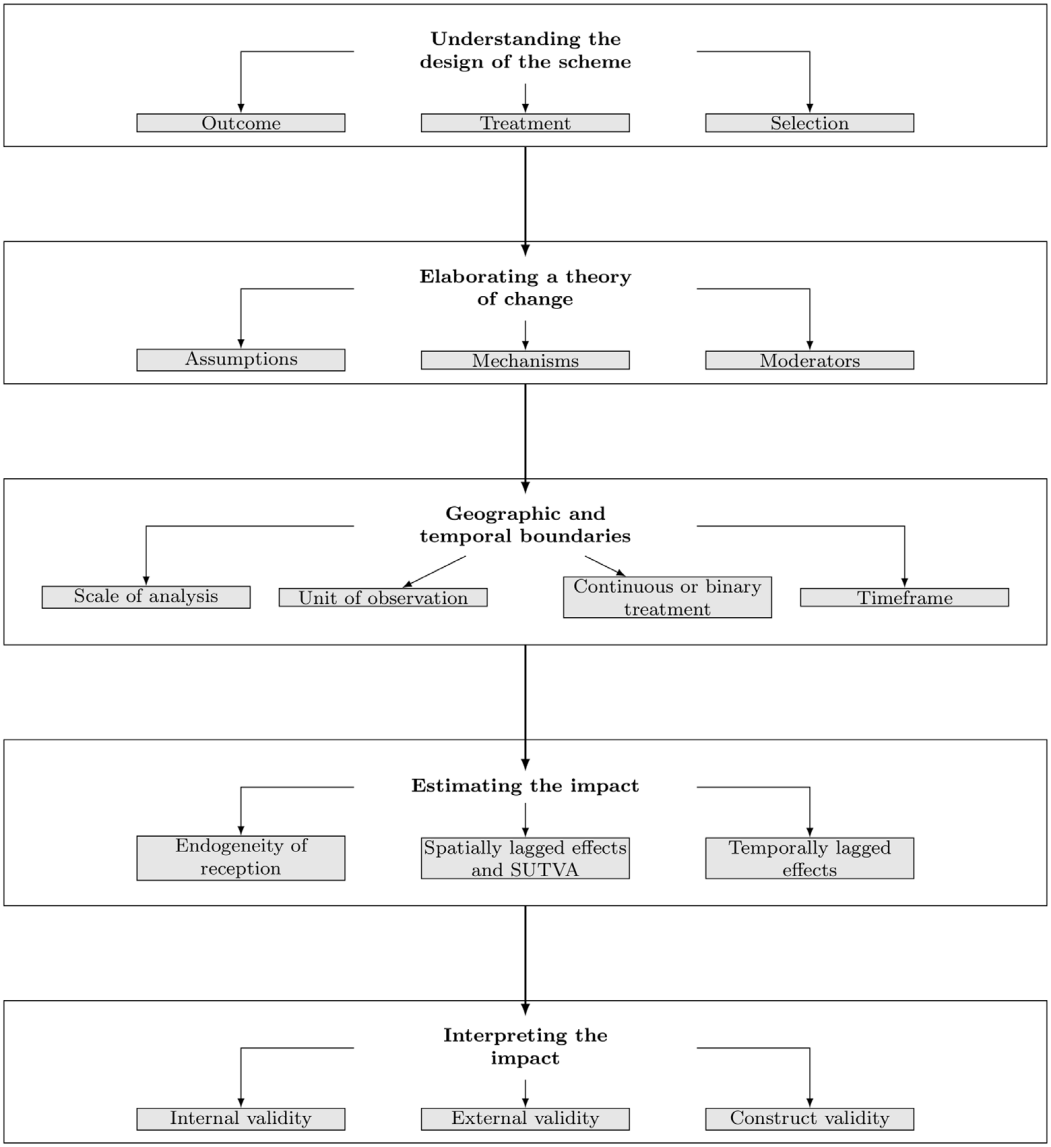
Design of an IE for PES.

However, when applying these methodologies in the context of PES programs, many adjustments need to be considered. We propose a framework that tracks the design of an IE from the elaboration of a theory of change to the final estimation. This article details the methodological procedure presented in Fig 1 and discusses each step of the IE method. Our aim is to propose guidelines and discuss methodological choices in designing IE for PES schemes (Fig 1).

To illustrate the main choices in adapting IE for PES schemes, we built on a systematic review of literature and used Google Scholar to search with the keywords “Impact evaluation” and “Payments for Environmental Services” (Consulted on the 23rd November 2015). We also searched for IE for other conservation instruments by associating “Impact evaluation” with “Protected Areas”, “Integrated Conservation and Development Programs” (ICDP), “Eco-certification”, “Agri-environmental policies” and “REDD”. Excluding citations, the total number of results for PES was 325. Searching for other conservation instruments, the total number of results increased to 2,540. To illustrate the most common methodological choices, we focus on articles that provide new evidence and aim at identifying a causal impact of PES schemes. The final sample of literature is composed of 51 studies presented in S1 Table. This sample may not be exhaustive, especially concerning unpublished studies; however we are confident that it is representative of existing literature.

## 2 Understanding the design of the scheme

The first step in designing an IE is to study the design of the PES scheme in order to understand its objectives and how it is implemented in the field.

### 2.1 Outcome and Treatment

Studying the scheme should allow us to identify both the definition of the treatment *T*_*i*_ and the outcome *Y*_*i*_ that is impacted by this treatment.

The main objective of any PES scheme is explicitly stated through the conditionality of the payments: avoided deforestation or degradation for forest conservation schemes or adoption of a sustainable practice for a practice-based program. These represent the main outcome variables *Y*_*i*_ but need to be transformed into a measurable outcome variable. Moreover, behind a single objective, there might be multiple measurable variables. For instance, if the payments are conditional on forest conservation, deforestation might be a restrictive indicator since degradation or forest fragmentation also matter [8, 9].

Unlike most conditional cash-transfer schemes, The objective of a PES program is not to benefit solely the payment beneficiary but also the buyer or any other person that could benefit from the provision of ecosystem services. As emphasized by Jack et al., (2008) [10] or Karsenty and Ezzine de Blas [11], many PES schemes rely on a proxy for the enhancement of ecosystem services. For example, payments can be conditional to the adoption of an agricultural technology but the main objective is to reduce soil erosion. Outcome variables *Y*_*i*_ are often defined according to these proxies (adoption of technology, deforestation, etc…). However, the biophysical aspects underlying the provision of ecosystem services should be studied. How does the proxy link to the ecological outcome [12]? In their study of Malagasy mangroves, Miteva et al. (2015) [13] extensively discuss the link between mangrove protection and carbon emissions.

Moreover, PES programs often have multiple objectives. For example, Sims et al., (2015) [14] highlight how a Mexican PES scheme tries to tackle both forest conservation and poverty alleviation. Focusing only on environmental objectives might not be relevant in such cases. Other objectives of the program should be considered as outcome variables for the IE [15].

Many other indirect effects can also be explored such as the impact of PES on economic activities and land use, governance, social capital and motivational aspects [16–19]. The impact of PES schemes on poverty has also been an important matter of concern in the literature [20–25]. These indirect effects are important not only to build empirical evidence but also because they can help validate a theory of change. One should consider which variables could be impacted by the program beyond its stated objectives and how to measure them.

Regarding the treatment, the term PES encompasses a large number of different interventions that one needs to disaggregate such as payments for reforestation, forest conservation and agroforestry. For instance, in their study of European Agro-Environmental Schemes, Chabé-Ferret et Subervie (2013) [26] identify five different modalities, each of which is considered as a different treatment. Moreover, as shown by Bauch et al. (2014) [27], a treatment may be allocated at the community level but within this community, households may or may not participate. Thus, different definitions of the treatment group can apply, from that of being in a community that receives the program to actively participating in the activity induced by the scheme or to receiving payments. The same applies for protected areas or community forests: the degree or type of protection can differ from one area to another and different treatments can be identified [28–32].

Therefore, based on a careful study of the implementation of the scheme, one must clearly identify the treatment and transform it into a variable such as *T*_*i*_ ≠ 0 if *i* is enrolled in a PES scheme. *Y*_*i*_ and *T*_*i*_ are the core of the IE but to evaluate the impact of *T*_*i*_ on *Y*_*i*_, the allocation of the treatment must be considered.

### 2.2 Selection process

As will be explained below, accounting for the differences between the treatment and the control group is a crucial issue for IE. Therefore, one must carefully study the selection process. Two types of actors in the selection process must be distinguished. On the beneficiary-side, since enrollment is voluntary, agents may have heterogeneous reasons for enrolling in the PES scheme, while on the buyer-side, the scheme designers often have targeting priorities.

On the beneficiary-side, the opportunity cost of enrollment has been acknowledged as a strong determinant of enrollment. For some agents, the opportunity cost of the enrollment is low or null because they already comply, at least partially, with the conditionality in the absence of payments [33, 34] and are hence more likely to enroll in a PES program. For example, agents that already conserve the forest are more likely to enroll in a PES program conditional on forest conservation. Opportunity costs must indeed be considered in the analysis. Although it may well be impossible to measure these opportunity costs, one can indirectly compute them, for example through average returns that could be obtained from clearing land.

Beyond opportunity costs, there are many other variables that influence enrollment in a PES. Structural factors such as transaction costs linked to remoteness from major markets, lack of access to information and poverty level are important determinants of enrollment [35]. At the household-level, risk aversion can also be a strong determinant since, contrary to agricultural crops, PES schemes offer a stable source of income that does not depend on climatic factors. Moreover, the cost of enrollment can be higher than the monetary opportunity costs. A growing body of literature looks at the motivational aspects behind enrollment in a PES scheme [36]. These can include intrinsic environmental motivations or trust in the agents implementing the scheme. If a PES scheme requires a change in agricultural practices or economic activities, some beneficiaries may be reluctant to adopt these practices for sociological reasons [37, 38]. Note that even if the PES allocation has been randomized among applicants, selection bias must be taken into account if one wants to extrapolate the results to the entire population of interest and not only to the population willing to join. Finally, it should be noted that, in most PES schemes, the beneficiaries do not need to enroll all of their plots in the program. The decision to enroll one area instead of another should also be studied.

Let’s now discuss the selection process on the buyer-side. The targeting priority of the actors involved in the design of the scheme must be studied. Some programs, such as national schemes, may have multiple official targeting criteria [14] which can include eligible areas, targeting index, differentiated payments etc… However, understanding the allocation mechanism often goes beyond studying the official allocation rules. Many actors, from the buyer to the beneficiaries of the payments influence the allocation process. There are many intermediaries in PES schemes that are likely to influence the allocation of payments. Therefore, allocation rules can change from one region to another [30, 39]. The same program may be interpreted, implemented and allocated differently in different areas and thus the factors influencing its allocation will not be the same.

On both the buyer and the beneficiary sides, these variables influence the allocation of PES schemes over both space and time. Therefore, they should be carefully analyzed and understood in order to accurately evaluate the impact of the PES program. As will be discussed below, studying these criteria should allow us to identify potential sources of endogeneity of the treatment.

## 3 Elaborating a theory of change

In the first step of the process, one must describe the scheme in order to evaluate its impact on a set of outcome. This second section discusses the elaboration of a theory of change that links the treatment *T*_*i*_ and the outcome *Y*_*i*_.

### 3.1 Hypotheses and mechanisms

*T*_*i*_ is supposed to impact *Y*_*i*_ but through which mechanisms? As emphasized by Miteva et al. (2012) [4], IE tells us *whether* and *where* a program had an impact but it is important to ask *why* and *how* it has (or not) an impact. In other words, as stated by Gertler et al. (2011, p22) [40], *≪ a theory of change is a description of how an intervention is supposed to deliver the desired results. It describes the causal logic of how and why a particular project, program, or policy will reach its intended outcomes ≫*. It involves understanding through which mechanisms the treatment impacts the outcome variable [28, 41].

Given our knowledge of a program’s design, one can assume that the program has an impact through a defined mechanism only if certain hypotheses are verified. For example, a PES for the adoption of a sustainable agricultural practice only has an impact if the beneficiaries would not have adopted the practice in the absence of payments. Therefore, one can hypothesize that the higher impact will be found in areas of higher poverty rates. What would the mechanisms be in that case? Poorer landowners can not bear the cost of adopting a new agricultural practice alone so the PES schemes help them to adopt the new practice. A crucial mechanism concerns agricultural trends. PES schemes often require a shift from an environmentally destructive economic activity to a more sustainable one. Therefore, the efficiency of the scheme relies on its ability to induce this shift. Studying the evolution over time of the economic activities of the program beneficiaries can help us to understand why the program did or didn’t have an impact.

There may be different explanations behind similar results. Based on the design of the PES scheme, one should ask what different mechanisms may be at play in order to be able to test them and understand why a program did or did not have an impact.

### 3.2 Moderators

Exploring impact heterogeneity can be very helpful in understanding the mechanisms in a given scheme. As emphasized by Miteva et al. (2012) [4] moderate impact does not necessarily mean no impact at all. Thus, it is important to investigate impact heterogeneity and, as highlighted by Ferraro and Miranda (2013) [41], *≪ go beyond average treatment effects ≫*.

Exploring heterogeneity according to defined moderators allows us to validate a theory of change, understand the mechanism, rule out rival explanations and make propositions to improve the effectiveness of the scheme. For example, if one hypothesizes that PES for sylvopasture is less likely to have an impact in areas with less cattle-ranching, studying the heterogeneity according to the prevalence of cattle-ranching seems relevant in testing a theory of change. If one hypothesizes that PES for forest conservation generates leakages in areas with high deforestation rates because no other livelihood options are available, testing heterogeneity according to a deforestation risk index or past deforestation could be useful. Heterogeneity can be explored through various methods including interactive variables, on subsamples or by estimating quantile treatment effects (i.e. treatment effects in different quantile of distribution of a given variable).

Many authors have studied impact heterogeneity in protected areas [42–46] but few have investigated this issue in the context of PES schemes. Thus far, impact heterogeneity has mainly been explored over time [47, 48], according to transaction costs or infrastructure [42] or according to a poverty index and geographic area [49]. Other important moderators including those related to the economic activity of the beneficiaries have received little attention so far. As explained by Corbera et al. (2009) [50], *≪ PES do not operate in a vacuum ≫*: other institutional factors matter in determining their efficiency. PES programs often interact with other policy instruments, such as regulatory environment or other subsidies [51] which modify the incentive structure. Agricultural subsidies or anti-poverty conditional cash transfers may have perverse effects on the forest cover [52, 53]. Another worthwhile investigation of the impact heterogeneity of PES schemes would be to examine the policy-mix defined as the different instruments in place that interact in a given landscape [15, 31, 54–56].

## 4 Designing the IE

Based on our understanding of the scheme and a theory of change, one should be able to design an IE. However, many issues arise and methodological choices must be made.

### 4.1 Scale of analysis

In the literature, some studies use a representative sample of all PES contracts. In such cases, geographic boundaries are the same for the PES and the IE. Examples are Alix-Garcia et al. (2012) [49] and Robalino and Pfaff (2010) [57], who studied the impact of a national PES scheme using a sample of beneficiaries and non-beneficiaries in Mexico and Costa Rica respectively. Other studies set boundaries by focusing on a specific zone at the regional or sub-regional level. Honey-Roses et al. (2011) [58], Costedoat et al. (2015) [59] and Arriagada et al. (2012) [60] also studied the impact of national PES (among other programs for Honey-Roses et al. (2011) [58]) but at the regional level in Mexico and Costa Rica. IEs of protected areas have even been implemented at the global level [61, 62].

The choice of a scale of analysis has consequences for the design of an IE. First if data are not available, the collection of necessary information for IE (surveys, remote sensing analysis etc…) is less costly for a smaller area even though remote sensing analysis with fine resolution is becoming increasingly available [63]. Second, as will be explained below, many estimators rely on the assumption that all variables affecting both treatment reception and the outcome are accounted for in the model. These variables are called confounding factors. In the case of PES, many confounding factors are geographic variables such as agro-ecological characteristics. The larger the area of study, the more heterogenous these characteristics will be, and estimations must take this heterogeneity into account, for instance, through within-strata matching or by including regional dummies in the estimation. Third, using a sample of all PES contracts automatically allows us to identify the average impact of the program on all beneficiaries, which is very useful in informing the process of policy design. Case studies at a lower level can be equally insightful but one must discuss the extrapolation of the conclusions of the IE to other areas.

Moreover, spatial spillovers, through leakage effects, are very likely to occur with PES [5]. Hence, beyond the group of beneficiaries and non-beneficiaries, boundaries of the study must be set in order to estimate leakages either by selecting a contiguous area or by including a buffer zone around PES contracts. Otherwise, estimates of the impact may remain partial because they are limited to the enrolled parcels.

### 4.2 Unit of observation

Once the boundaries of the study area are set, one must determine the relevant unit of observation *i* given the outcome variable. This is crucial because, to comply with the assumption of the estimators highlighted below, the observations must be independent. Moreover, one should be able to accurately measure the confounding factors for each of them.

Units of observation include pixels [57], grids [59], plots or parcels [49, 58] and decision-makers [26, 60]. We will here consider that, if the study is at the decision-maker level, the unit of observation includes all parcels managed by the same agent. The difference between pixels and grids is that pixels are randomly chosen from the remote sensing analysis while grids or polygons encompass a various number of pixels (for instance all pixels within each cell of 500m x 500m). A plot of land or a parcel, contrary to pixels, is not defined through a grid but according to economic variables (for instance, the area under PES enrollment, the agricultural fields or contiguous areas with homogeneous land use).

Ideally, the unit of observation must be homogeneous regarding:

- Treatment status and exposure
- Land tenure
- Size of the units of observation
- Confounding factors within this unit of observation

If the unit of observation contains both enrolled and unenrolled land, it may be impossible to identify the impact if leakages occur. The impact on enrolled and unenrolled areas (leakages) can not be disentangled [64]. For example, let’s consider a unit of observation that contains both forests enrolled and not enrolled in a PES program for forest conservation. The program impact can be null if the enrolled forests have been deforested which suggests a lack of monitoring or that deforestation has been displaced to unenrolled areas.

A unit of observation should not overlap between two different landowners. Many confounding factors such as land endowment or economic activities are computed at the landowner level, and many spatial variables, such as distance to agricultural fields for conservation PES schemes, must be in comparison to other parcels owned by the same landowners. In the case of deforestation, the relative opportunity costs of a parcel of forest compared to other parcels owned by the same landowner matters more than its absolute value [65]. Attention must also be paid to land tenure if leakages are likely to occur. Leakages through activity shifting are very likely to occur if the beneficiaries do not enroll all of their parcel in the scheme so it is crucial to link the land to the landowner.

Taking into account homogeneity of size of the area within the unit of observation is important in order not to compare apples with oranges. Moreover, if the outcome variable is the variation of land-use, such as deforestation rates, this outcome may depend on the size of the unit of observation. A 4% decrease in deforestation rates indicates a different impact when dealing with an observation of 25ha than with 200ha.

The final condition mentioned above is the homogeneity of confounding factors. In previous sections, we highlighted that a careful study of the selection process is crucial to the design of the IE. By averaging variables at the plot-level or through gridding, one may fail to consider heterogeneity of agro-ecological characteristics such as soil condition. If these characteristics are part of the confounding factors, it will not be possible to account for this heterogeneity in the estimation and results will be biased

Using a method based on a random selection of pixels complies with all of these criteria and makes it possible to accurately account for any geographic heterogeneity in the area under protection. However, as highlighted by Miteva et al. (2015) [13], assigning socioeconomic characteristics to pixels might introduce various problems including the issue of pseudo-replication and auto-correlation for spatially close pixels (many pixels will have the same socioeconomic characteristics). Moreover, landowners make decisions on plots of land and not on pixels. Therefore, one may feel more comfortable selecting areas that are relevant for decision-making as parcels or gridding.

### 4.3 Continuous or binary treatment

The previous sections allowed us to identify the unit of analysis and how the treatment group is defined. However, there are still different ways to define a treatment group. PES programs are usually modeled using dummies. Protection through PES or Protected Areas is a binary decision so most programs are usually modeled using dummies. However, protection can be heterogeneous with respect to the time spent in the program, the payments received and the area protected, one can model a PES scheme as a continuous variable.

Choosing a continuous treatment changes the way of dealing with the selection bias. The concern here is not only the enrollment in the scheme but also the order of entry into the program or the percentage of land enrolled in the program. Estimators that deal with endogeneous continuous treatments exist (two-stage least squares [21, 43] or generalized propensity scores [64] for instance) but it may be impossible to account for endogeneity in both treatment reception and heterogeneity.

It is often not possible to take into account the endogeneity of both selection into the program and treatment exposure. If all beneficiaries receive similar payments and enroll for a similar number of years, one can model reception as a dummy variable and only consider the selection issue. However, if the exposure to the treatment is very heterogeneous, modeling reception as a dummy fails to take this heterogeneity into consideration. In such a case, if the proportion of non-beneficiaries is high, it seems crucial to deal with the endogeneity of treatment reception through a pre-matching [66] or by using an approach based on control functions [67] before estimating the model with a continuous treatment. By contrast, if there are few non-beneficiaries, one should focus on endogeneity of treatment exposure.

### 4.4 Timeframe

Our last concern in designing an IE is the timeframe. PES schemes are usually fixed-term contracts but the relevant timeframe for an IE may be longer than the length of the contract.

First, some landowners may anticipate the onset of the program and change their behavior before enrolling in a PES scheme. For forest conservation schemes, forest owners can stop clearing in order to send a signal of good behavior to the authorities. A contrary hypothesis is that forest owners will anticipate the future ban on converting land by cutting more trees before the ban becomes effective. Moreover, if the amount of payments is a function of a baseline, it may be tempting to increase deforestation to influence the baseline [2].

PES programs may also have lagged effects: they can help to finance a transition to non-degrading activities and permanently impact the provision of environmental services [68, 69]. On the other hand, if landowners comply with the program’s conditionality during the contract, they can get back to a business-as-usual situation after the program ends. A PES program can even have negative effects if it relaxes a liquidity constraint which leads to more environmental degradation after the end of the program [70].

PES schemes may thus have both positive or negative ex-ante and ex-post effects on forest cover. The direction of the bias is theoretically unknown and can also vary from one beneficiary to another. However, the lagged effects of PES programs are crucial for both identification of a causal effect, as will be discussed below, and for permanence of a given scheme’s impact. In order to evaluate the magnitude of such an effect, future and former beneficiaries should be considered separately in the analysis and not be included in the control group. As a matter of fact, if a relevant control group exists and data allows it, it is possible to estimate the lagged impact of PES on these cohorts of beneficiaries before and after the treatment.

## 5 Estimating the impact

Once the boundaries of the IE have been set, one should consider how to identify the impact of the treatment on the outcome. This includes choosing the appropriate estimator and dealing with the main assumptions that allow us to estimate the impact without bias.

### 5.1 Endogeneity of the reception

Remember that IE aims at evaluating the causal effect of the program beyond simple correlation: there may be rival explanations for a result [71]. If these rival explanations cannot be ruled out, the estimates may be attributed to a simple correlation between two variables.

Endogeneity of PES reception is the main issue for an IE. Endogeneity mainly arises because of confounding factors *X*_*i*_ conjointly influencing the treatment *T*_*i*_ and the outcome variable *Y*_*i*_. The previous sections emphasized that PES beneficiaries are likely to be very different from non-beneficiaries. It is highly possible that these differences also impact the outcome variable. For instance, if the program succeeds in targeting hot spots of deforestation then the enrolled areas have higher deforestation rates. On the contrary, if because of self-selection, the beneficiaries are less dependent on the forest cover, they will have lower deforestation rates. In both cases, one must deal with endogeneity of the PES reception.

Based on the study of the selection process, one should be able to identify these confounding factors. If the PES scheme has been randomized across the population of interest, one must check whether the randomization succeeded in balancing observable covariates. Then the impact can easily be estimated through a mean-comparison test or by using ordinary least squares. To date, Randomized Controlled Trials have been poorly used in the context of PES. However, on a small scale, randomized experiments hve allowed authors to investigate the role of social preference [41, 72] or local leadership [73] in the provision of environmental services.

If the treatment is not randomized, the type of intervention and the selection process influences the variables included in *X*_*i*_. Structural factors of environmental issues should be considered. In the case of deforestation, these will include covariates such as slopes and elevation, population density or remoteness from the main markets, transaction costs and agricultural terms of trade [74]. If the scheme is allocated to households, confounding factors may include the household size, household head characteristics, the type of economic activities they rely on or their land endowment. At the community-level, other covariates related to collective action or organizational capacities should also be considered.

Some of the covariates are observable variables and can be directly accounted for in the estimation. However, some confounding factors are difficult, if not impossible, to measure. These unobservable variables include the intrinsic motivation to enroll in an environmental scheme, the environmental consciousness and agro-ecological characteristics of an area (soil conditions etc…). How do we account for these variables?

Matching methods rely on a crucial assumption called unconfoundedness. As emphasized in Eq 1, to identify the impact of the treatment *T*_*i*_, all counfounding factors must be accounted for in the estimation.

$$Y_{i}⊥T_{i}|X_{i}$$ (1)

This statement makes it clear that the choice of a control group is crucial in controlling for unobservable covariates. As emphasized above, the choice of the control group must be based on a careful study of the allocation mechanism. Among many other examples, Alix Garcia et al. (2012) [49] used applicants who were rejected from a PES program due to insufficient funding to control for the willingness to join the program, while Arriagada et al. (2012) [60] used landowners located in a buffer zone around areas under PES contracts to ensure that the control group had similar agro-ecological characteristics.

The choice of a matching technique must be adapted to the context of the studies and the available data [75, 76] and appropriate tests provided [20, 77]. Moreover, if the impact is estimated through matching, observable confounding factors *X*_*i*_ should be measured at the baseline year, i.e. before treatment implementation while *Y*_*i*_ is the outcome after PES implementation. As a matter of fact, the PES scheme may impact those covariates (economic activities for example) which would bias the matching procedure. Note that matching methods have often been used as a pre-process to select a control group before estimating the impact through various econometric models according to the type of outcome variable or the research interests [49, 77–80]. This might be especially convenient if the outcome is censored or binomial.

It should not be forgotten that matching is aimed at balancing covariates between two groups and cannot be used to identify individual pairs of observations. For instance, if a PES is assigned to three landowners, identifying three control landowners through matching will not balance covariates between the two groups and it will not be possible to identify the impact of the program. If both groups are large enough, matching methods can weight the observations in the control group to build a counterfactual that is statistically similar to the treatment group. The difference between individual pairs of observations cannot be interpreted independently of the rest of the distribution. Moreover, if the intervention is at the village level, most of the confounding factors are measured at the village level and investigating many households in only a few villages will not solve this issue. However, if panel data with a large enough temporal dimension are available for these few treated villages and a control group, it remains possible to estimate the impact through synthetic control [81, 82].

Compared to matching estimations, Difference-in-difference (DID), that has also been labeled as Before-After-Control-Intervention [83–85], controls for time-unvarying unobservable confounding factors. The main assumption of DID is called *parallel trend*. It states that there are no time-varying confounding factors affecting both reception of the treatment and the outcome variables. Moreover, time-varying observable covariates can also be accounted for using panel data [79, 86–88]. Therefore, the assumption of DID are less restrictive than for matching, so DID should be favored if data are available. Note that DID can also be implemented as a difference of variation rates. In that case, the estimator is called triple difference and captures the difference between the variation of the outcome variable before and after the program implementation. In other words, it indicates if the program increased or decreased the variation rate of the outcome variable [26, 89].

The choice of a relevant control group remains important. As a matter of fact, DID allows one to identify the impact of the treatment if there are no time-varying unobservable confounding factors. For instance, if after the implementation of the PES program, the control group is affected by a climatic shock that increases the adoption of a sustainable practice, the parallel trend assumption does not hold.

Note that both methods can be combined through DID-matching [26, 90]. This method matches observations based on observable covariates measured before the implementation of the treatment but the outcome variable is not *Y*_*i*_ but rather *ΔY*_*i*_, the variation of the outcome after implementation of the treatment. As with DID, it controls for time-unvarying unobservable covariates. In this case, the unconfoundedness assumption is less restrictive than for the matching estimator and is given by Eq 2:

$$\Delta Y_{i}⊥T_{i}|X_{i}$$ (2)

Finally, instrumental variable methods such as control function methods or two-stage least squares relax the assumptions on unobservable covariates. However, to be considered as valid instruments, the variables must be strong predictors of the treatment without directly impacting the outcomes. Weak instruments that are only poorly correlated with the treatment should not be used since they may introduce more bias in the estimation [91]. Therefore, there are very few examples of exogeneous instruments [21, 43, 92]. Given the discussion of confounding factors above, it is easy to understand that most of the determinants of the treatment also impact the outcome variable. Moreover, as will be emphasized below, these methods identify a Local Average Treatment Effect (LATE), which might be less relevant for decision-making.

The choice of the unit of observation also has consequences for the choice of an estimator. For example, deforestation is binary (forest or not forest) at the pixel-level but continuous at the parcel level (deforestation rate or number of hectares lost). If the outcome is binary, the type of estimator might be different and probit or logit estimations are preferable to ordinary least squares or two-stages least squares that do not take into consideration the discrete nature of the outcome variable.

All of these methods can be implemented with continuous treatment. Studying the impact with continuous treatment may even be the only relevant approach if there is no appropriate control group. Let us consider a watershed where all landowners are accepted for a PES program. The only landowners who are not in the program are likely to be structurally different from the others and will thus not constitute a relevant control group. Looking for a hypothetical control group inside or outside this watershed is bound to fail as important unobservable confounding factors will not be taken into consideration.

Note that, we focus here on the main types of estimators used in the literature, but other estimators such as pipeline regression or regression discontinuity design can also be used if the context allows [7, 93].

### 5.2 Spatially lagged effects and SUTVA

All of the estimators described above rely on the Stable Unit of Treatment Value Assumption (SUTVA). This assumption states that there are no diffusion effects of the treatment i.e. that the outcome of one individual is only influenced by the individual’s own status in relation to the treatment. If SUTVA is verified, the fact that *j* ≠ *i* receives the program (or not) does not impact the outcome *Y*_*i*_.

In the context of conservation instruments, validating SUTVA automatically requires assumptions about the leakage effects of a given program. One must question if leakages are likely to occur given the design of the scheme and how it will affect the control group. For instance, for conservation PES, if the unprotected parcels are impacted by the treatment, SUTVA does not hold. The same can be true with practice-based PES if the practice is generalized in non treated parcels that don’t receive PES. In both cases, the control group is influenced by the treatment and does not constitute a relevant counterfactual.

One solution would be to remove the buffer zone from the control group [58]. Under the assumption that no secondary leakages have occurred due to market equilibrium effects, removing the buffer zone makes it possible to correctly identify the impact. Nevertheless, this only captures the impact of the program on enrolled parcels, while total estimates of additionality should also include leakages. Therefore, leakages should be estimated either through matching [49, 58] or using a spatially lagged variables [80].

But where should we look for leakages? A theory of change and a good understanding of the program’s design and its implementation should help to answer this question. As highlighted by Aukland et al. (2003) [94] in the case of avoided deforestation, some factors such as the availability of alternative livelihoods options or the engagement of the beneficiaries in commercial activities can influence the emergence of leakages. For example, if enrollment implies a switch by beneficiaries to activities that are less dependent on the forest cover, leakages are likely to occur if these activities are not available. However, if the beneficiaries are not involved in commercial activities, one should not expect leakages through market effects [95] but only through activity shifting. Moreover, if non-enrolled forests land near the enrolled forests is easily available to the beneficiaries, the leakages are likely to occur in these areas.

SUTVA may also not hold if the treatment changes the behavior of the control group. A change of behavior can be attributable to various mechanisms. A widely-studied phenomenon in IE is the John Henry Effects which refers to a change in behavior of the control group in an attempt to compensate for their disadvantage compared to the treated group. For example, the control group for a PES reforestation program may decide to reforest in order to claim for payments. Moreover, a growing body of literature has addressed crowding-in and crowding-out effects [36]. This literature suggests that remunerating certain landowners changes their intrinsic environmental motivations. This literature suggests that economic incentives, such as PES schemes, can reinforce (crowd in) or undermine (crowd out) the commitment toward nature conservation. If these effects arise, the treatment impacts the behavior of the control group and it can not be considered as a relevant counterfactual since SUTVA does not hold. Controlling for these effects in the estimation is complicated. If the intervention is a randomized control trial, it is possible to mitigate diffusion effects through a transparent allocation procedure [96]. In other cases, qualitative work should drive the choice of a control group in order to avoid allowing motivational aspects to bias the IE. Moreover, as for leakages, it would be very insightful to explore the extent of behavioral changes by analyzing the effect of the treatment on the non-beneficiary population that is subject to motivational changes.

### 5.3 Temporally lagged effects

Eventually, another crucial assumption is the absence of Ashenfelter’s dip [97]. This assumption is not verified if transitory shocks affect the control and treatment group differently before treatment implementation. If shocks affect pre-treatment characteristics, the estimators are biased.

One factor that could lead to Ashenfelter’s dip is the anticipation of the treatment reception. DID or matching often use pre-treatment information to control for differences between the control and treatment group before the PES scheme implementation. Land use prior to the program is usually considered to be exogenous but if the agents anticipate the program, this hypothesis cannot be validated. If the pre-treatment characteristics have already been impacted by the treatment, we face an Ashenfelter’s dip and the direction of the anticipation effect is unknown.

Therefore, one should ensure that this effect does not threaten the IE through conducting qualitative interviews. Using a longer timeframe can also help to overcome this problem. If the effect is very likely to occur, it might be more relevant to control for pre-treatment values of the covariates, not just shortly before the treatment implementation but rather a few years before. Note that if the data for *X*_*i*_ and *Y*_*i*_ exist for a number of years before the program, it could be possible to estimate the anticipation effect using the usual techniques presented above. Moreover, Abadie (2005) [98] proposes estimators that deal with Ashenfelter’s dip in the case of DID estimations.

## 6 Interpreting the impact

Once the impact is estimated, one must characterize it according to its internal, external and construct validity.

### 6.1 Internal validity

Internal validity is the minimum one should question regarding the relevance of the estimates. As explained by Ravallion (2007) [7], internal validity concerns the ability to obtain a “*reliable estimate of the counterfactual outcome in the specific context*”.

The internal validity of the estimation depends on the type of estimators and the geographic boundaries. If the treatment has been randomized amongst a large population, one identifies an Average Treatment Effect (ATE) as given in Eq 3 with $Y_{i}^{T}$ the outcome with the treatment and $Y_{i}^{NT}$ the outcome without the treatment.

$$ATE=E(Y_{i}^{T}-Y_{i}^{NT})$$ (3)

The ATE does not depend on the status of the beneficiaries *T*_*i*_ and we can hypothesize that the impact would have been the same if the control group had received the treatment.

Matching or DID identifies an Average Treatment Effect on the Treated (ATT).

$$ATT=E(Y_{i}^{T}|\left(T_{i}=1\right)-Y_{i}^{NT}|\left(T_{i}=1\right))$$ (4)

The internal validity of the ATT is limited to the beneficiaries with *T*_*i*_ = 1 as shown in Eq 4. If the assumptions of the estimator are verified, the impact is well identified, but one cannot hypothesize that the impact would have been the same for the non-beneficiaries.

Instrumental variables methods estimate a LATE. The internal validity of the LATE is limited to observations for which the instruments *Z*_*i*_ influence the reception of the program *D*_*i*_ as shown in Eq 5 [99].

$$LATE=E(\left(Y_{i}^{T}-Y_{i}^{NT}\right)|T_{i}\left(Z_{i}=z\right)\neq T_{i}\left(Z_{i}=w\right))$$ (5)

The extent of the internal validity of the LATE is likely to be limited to a sometimes small percentage of the population of interest and may be of less interest to decision makers than an ATT [100, 101]. However, the above section about endogeneity of the treatment variable show that it may be very difficult to comply with the assumption of matching and DID estimators and that the estimates obtained are likely to be partially biased. If exogenous variables that strongly impact the reception of the treatment are available, one should privilege estimators using instrumental variables instead of matching or DID. However, if those instruments are only poor predictors of the treatment, the coefficients estimated are likely to be biased [91] and the internal validity of the estimates is very limited.

### 6.2 External and construct validity

External validity concerns the extrapolation of the results in other settings for another population or in other places. In Ravallion (2009)’s words, external validity is the: ≪ degree of generalizability ≫ [102]. It is often discussed as the possibility to extrapolate the results over space, to new areas or beneficiaries, but generalization over time should not be forgotten. Indeed, even for the same beneficiaries, the impact could be different in another period [103].

First, one should discuss the statistical representativity of the sample used for IE. If the sample used for the IE is statistically representative of a population of interest, external validity is likely to be large. If the sample corresponds to a very specific share of the population, little can be said about external validity. If data are available, simple statistical tests on observable covariates *X*_*i*_ should help to answer this question.

Second, a theory of change should be helpful in understanding the generalizability of the results. As highlighted above, this theory states the hypotheses and mechanisms that lead to the estimated impact. Therefore, it provides a framework under which external validity must be discussed. Are these hypotheses likely to be validated if the program is allocated to other beneficiaries or at another time? Are the mechanisms likely to be the same? A good knowledge of the context and the region of study are essential in answering these questions. For example, if a PES scheme for forest conservation has been evaluated in an area of high deforestation rates, the results regarding additionality are likely to be different in an area with small deforestation rates. By contrast, if one estimates that the scheme generates leakages through a given mechanism, for instance through activity shifting, it is possible to discuss the emergence of leakages in another area given the design of the scheme, the land tenure system or the type of economic activities involved [94]. Generalizability is especially important for pilot-projects. Pilot-projects are often implemented to test whether or not the program is effective before scaling-up. However, these pilot projects may benefit from more attention from the designers and are implemented in very specific areas. Will the program have the same impact if the designers and the evaluators are less present in the day-to-day implementation? Discussing external validity through a theory of changes should help to answer these questions.

A last concern is construct validity or the degree to which an IE measures what it claims to be measuring. Remember the objective of PES is to generate incentives for the sustainable management of natural resources to enhance the provision of environmental services. IE allows one to compute the additionality of the program but does not tell us much about the way it impacts the institutions that manage the forest cover. Neither does it tell us what the situation would be if the payments stop in the future. Therefore, one should be careful in interpreting the results of an IE and in discussing its implications for the provision of ecosystem services in the long-term.

## 7 Conclusion: Future research and policy implications

This article proposes a framework for PES IE by tracking the various steps in the evaluation process, from the analysis of the scheme design to the interpretation of the results. It emphasizes the questions one should ask when trying to estimate a causal effect of a PES scheme. Through this process, we also show that qualitative work to understand the context of the study and build a relevant theory of change is crucial for IE. We acknowledge that designing an IE that addresses all the issues highlighted here may be impossible either because of lack of data availability or because the context of the study does not allow us to estimate an unbiased impact. However, one should be aware of the various choices at each step of the estimation in order to deal with the biases as effectively as possible and to exclude rival explanations.

The literature that looks at the impact of nature conservation policies is rapidly growing but many research areas still need to be explored. As highlighted by many authors [4, 5, 104], more attention should be given to elaborating theories of change that would allow a more comprehensive understanding of *why* and *how* policies are effective or not. This is particularly important given the keen interest of policy-makers for so-called market-based instruments [105]. The number of PES schemes is likely to keep growing and little is known of the external validity of the existing evidence. Theories of change should help to overcome this issue and inform the implementation of new PES schemes. In addition, more attention should be given to agricultural trends. Beside timber production, agricultural activities are the main determinants of land use change. Focusing IE on the environmental outcomes without considering the underlying causes of deforestation is a restrictive approach to understand the impact of the program. Third, little empirical evidence exists about the long-term impact of the schemes. A growing literature discusses the effect of PES programs on intrinsic environmental motivations [36]. These behavioral aspects influence the permanence of PES impact and its effectiveness in the long-run. Some PES schemes have now been implemented for long enough to enable us to look at lagged impacts and permanence, especially if the payments end, is becoming a crucial area of research for IE.

Finally, IE aims at identifying the additionality of PES, but what is the additionality of IE? It should not be forgotten that IE needs to inform the public debate and the policy making process. As highlighted by Ravallion (2009) [102], the research community may have a tendency *≪ to (on the one hand) encourage serious effort to assure the internal validity of a piece of research within its predetermined scope, but (on the other hand) to be quite relaxed about the claims made about implications for policy or relevance to other settings ≫*. Craigie et al. (2015) [106] recently discussed the reasons why IE of conservation instruments may fail to make a difference. These reasons include the fear of exposing failures or technical and economic barriers to the generalization of IE. The authors also show that IE is sometimes perceived as a costly and time-consuming activity to build knowledge that already exists. To address these concerns, IE must provide rigorous evidence on the impact of the schemes and mechanisms but also emphasize how these results can be useful to decision-makers to improve the effectiveness of funds allocated to conservation.

## Supporting Information

S1 Table Impact evaluations of conservation instruments. (PDF)
