# Supplementary material for: Evaluating Payments for Environmental Services: Methodological Challenges
Source: PLoS One. 2016 Feb 24;11(2):e0149374. doi: 10.1371/journal.pone.0149374 (PMC4766196; doi:10.1371/journal.pone.0149374)
Supplement: S1 Table — (PDF) [file pone.0149374.s001.pdf]

Table 1. Impact evaluations of conservation instruments

Authors	Instrument	Country
Ahmadia et al. (2015)	Protected Areas	Indonesia
Alix-Garcia et al. (2012)	PES	Mexico
Alix-Garcia et al. (2015)	PES	Mexico
Amin et al. (2014)	Protected Areas	Brazil
Andam et al. (2008)	Protected Areas	Costa Rica
Andam et al. (2010)	Protected Areas	Costa Rica and Thailand
Arriagada (2008)	PES	Costa Rica
Arriagada et al. (2012)	PES	Costa Rica
Bauch et al. (2014)	ICDP	Brazil
Bruggeman et al. (2015)	Zoning policy	Cameroon
Busch et al. (2015)	Moratium	Indonesia
Canavire-Bacarreza and Hanauer (2012)	Protected Areas	Bolivia
Caplow et al. (2011)	REDD+ Projects	Brazil, Bolivia and Belize
Chabé-Feret and Subervie (2013)	Agri-environmental Measures	France
Cisneros et al. (2015)	Blacklisting	Brazil
Clements and Milner-Gulland (2015)	PES<br><br>protected Areas	Cambodia
Clements et al. (2013)	PES	Cambodia
Clements et al. (2014)	Protected Areas	Cambodia
Costedoat et al. (2015)	PES	Mexico
Cuenca et al. (2015)	Protected Areas	Ecuador
Darling (2015)	Protected Areas	Kenya
Ferraro et al. (2011)	Protected Areas	Costa Rica and Thailand
Ferraro et al. (2013)	Protected Areas	Bolivia, Costa Rica, Indonesia and Thailand
Haruna et al. (2014)	Protected Areas	Panama
Honey-Roses at al. (2011)	PES<br>Protected Areas	Mexico

Authors	Instrument	Country
Jack (2009)	PES	Kenya
Jack and Recalde (2015)	PES	Bolivia
Joppa and Pfaff (2010)	Protected Areas	Global
Le Velly et al. (2015)	PES	Mexico
Miranda et al. (2016)	Protected Areas	Peru
Miteva et al. (2015)a	FSC Certification	Indonesia
Miteva et al. (2015)b	PES	Indonesia
Nelson and Chomitz (2009)	Protected Areas	Global
Pfaff et al. (2008)	PES	Costa Rica
Pfaff et al. (2009)	Protected Areas	Costa Rica
Pfaff et al. (2014)a	Protected Areas	Mexico
Pfaff et al. (2014)b	Protected Areas	Brazil
Pfaff et al. (2015)a	Protected Areas	Brazil
Pfaff et al. (2015)b	Protected Areas	Brazil
Rasolofoson et al. (2015)	Community forests management	Madagascar
Robalino and al (2015)	PES<br>Protected Areas	Costa Rica
Robalino and Pfaff (2013)	PES	Costa Rica
Robalino et al. (2008)	PES	Costa Rica
Robalino et al. (2014)	PES	Costa Rica
Rueda et al. (2015)	Eco-certification	Colombia
Sauer et al. (2012)	Agri-environmental Measures	United Kingdom
Sharma et al. (2015)	REDD+ Projects	Nepal
Sills et al. (2015)	Mapping, monitoring and Alternative activities	Brazil
Simonet et al. (2015)	REDD+ Projects	Brazil
Sims (2010)	Protected Areas	Thailand
Sims et al. (2014)	Protected Areas	Thailand
Takahashi and Todo (2012)	Community forests management	Ethiopia
Wendland et al. (2015)	Protected Areas	Russia
Yanez-Pagans et al. (2013)	PES	Mexico
